# Influence of conflicting prior information on action anticipation in soccer players: an ERP study

**DOI:** 10.3389/fnbeh.2023.1320900

**Published:** 2023-12-07

**Authors:** Qingchun Ji, Chenglin Zhou, Yingying Wang

**Affiliations:** ^1^Department of Physical Education, Shanghai University of Engineering Science, Shanghai, China; ^2^Sports Economic Management Research Center, Shanghai University of Engineering Science, Shanghai, China; ^3^School of Psychology, Shanghai University of Sport, Shanghai, China; ^4^Key Laboratory of Motor Cognitive Assessment and Regulation, Shanghai University of Sport, Shanghai, China

**Keywords:** prior information, soccer players, action anticipation, key time points, cognitive processing

## Abstract

**Objective:**

Prior probability information and visual kinematic information are essential for action anticipation in athletes. The aims of this study were to examine how conflicting prior information influences anticipatory judgment in athletes vs. non-athletes and to explore the underlying cognitive mechanisms.

**Methods:**

The aim of Experiment 1 was to determine the moment when prior information influenced action anticipation in athletes vs. non-athletes. To that end, 17 semi-elite soccer goalkeepers and 18 non-athletes received prior information about the probability of the direction that a player on a video would kick a ball into the goal. Participants then anticipated the trajectory of the ball when the action of the player’s kick on the video was truncated at the moment the foot contacted the ball (time T) or one frame (T-1; 50 ms) or two frames (T-2; 100 ms) before the foot-ball contact. The aim of Experiment 2 was to elucidate the adaptive cognitive-motor behavior exhibited by highly trained soccer players at the moment when their anticipatory performance was most influenced by prior information. Experiment 2 included 27 different semi-elite soccer players with many years of experience as a goalkeeper and 27 different non-athletes. Participants anticipated the direction of the kick when the kinematic action of the kicker at the moment the anticipatory performance of the participants was most influenced by prior information (as determined in Experiment 1) was congruent, incongruent, or neutral. Action anticipation accuracy and response time were evaluated for both experiments, whereas event-related potential components N1, N2, and P3 were assessed only in Experiment 2.

**Results:**

The results of Experiment 1 showed that anticipatory accuracy was significantly higher among athletes than non-athletes and that anticipatory accuracy with directional information given was significantly higher than that when no prior information was given or when prior information without directional information was given (*p* < 0.001) for both T-1 (*p*’s ≤ 0.034) and T-2 (*p*’s < 0.001) occlusion points. In Experiment 2 using those two video occlusion times, the amplitude of the N1 component, which reflects selective attention to stimulus properties, was significantly higher in athletes than in non-athletes (*p* < 0.001). The amplitude of the N2 component, which has been associated with conflict monitoring, for the incongruent condition was significantly higher than that for both neutral (*p* < 0.001) and congruent (*p* < 0.001) conditions in athletes. Non-athletes exhibited no significant N2 amplitude differences for any prior information condition.

**Conclusion:**

Integrating prior information enhanced action anticipation in semi-elite soccer players, particularly 50 and 100 ms before the foot-ball contact. Semi-elite soccer players prioritized early selective attention and conflict monitoring of kinematic information, facilitating action anticipation using the prior information.

## 1 Introduction

In the realm of competitive ball sports, athletes must swiftly react to their opponents’ actions and to moving balls within tight time constraints. However, inherent biological constraints, such as delayed neural transmission, can impede optimal response decision-making if athletes wait to process all the available visual information. For instance, in the context of shooting a soccer ball, goalkeepers perform swift ball-saving movements in a specific direction to enhance their likelihood of catching the ball and preventing a goal (Kang et al., 2022). Dicks et al. (2010) found that goalkeepers exhibited earlier initiation of movement responses when presented with consecutive visual cues from a penalty taker’s actions. During this critical moment, the information about the shooting motion of the opposing player may not be fully revealed. As outlined in Hogendoorn’s “time perception” mechanism proposed in 2021, individuals tend to anticipate forthcoming events in order to compensate for the delay in receiving complete kinematic information at any given moment (Hogendoorn, 2022). Consequently, anticipatory judgments rely on presently incomplete information, such as whether the goalkeeper successfully saves the ball, contingent on the precision of early predictions regarding the direction of the incoming ball.

Relying solely on current kinematic information for action anticipation carries relatively high decision-making risks. Athletes often combine additional information stemming from anticipation and enhance the efficiency of action anticipation to mitigate response losses (Thomas et al., 2022). For instance, during the quarterfinals of the 2006 FIFA World Cup, Germany faced Argentina, and the German goalkeeper received a note from the coach summarizing the typical past shooting directions of the opposing player. Leveraging this information, the goalkeeper successfully predicted the shooting directions of two goals, ultimately leading to Germany’s victory. Körding (2007) applied Bayesian statistical theory to competitive sports scenarios, suggesting that athletes comprehensively consider predicted action outcomes based on kinematic information and prior information during action anticipation before making response decisions (Körding, 2007). Compared with relying solely on kinematic information for action anticipation, athletes can achieve higher efficiency and experience fewer losses in fast-paced and interactive sports competitions when they combine prior information with kinematic data (Wang and Chu, 2021). In recent years, numerous studies have investigated how prior information impacts athletes’ action anticipation performance (Mann et al., 2014; Gredin et al., 2018; Broadbent et al., 2019; Gredin et al., 2021). These findings consistently demonstrate that advance knowledge, such as opponents’ action tendencies, the probability of action outcomes, and commonly employed technical and tactical strategies, significantly enhances athletes’ action anticipation performance. As proposed by Williams and Jackson in 2019, future research should focus on exploring the impact of prior contextual information on action anticipation and its role in practical sports training (Williams and Jackson, 2019).

The “time perception” mechanism posits that action anticipation can occur at any point during the event process (Hogendoorn, 2022). As the event unfolds, the accumulation of current kinematic information gradually leads to the complete presentation of action-related data. This process may influence the extent to which reliance on action anticipation depends on both kinematic and prior information. Illustrating this concept with cricket batting, Runswick et al. (2018) investigated the interplay between kinematic and prior information during the anticipation of deliveries. They employed a temporal occlusion paradigm to segment videos of deliveries from bowlers into four time points relative to ball release (pre-run, mid-run, pre-release, and post-release). The findings revealed that skilled cricket batters primarily consider kinematic information to be more crucial at the pre-release moment, while they consistently utilize prior information throughout the anticipation process (Runswick et al., 2018). This implies that the integration of kinematic and prior information exhibits a temporal characteristic. One issue stemming from this implication is the necessity to ascertain, within the action anticipation process as kinematic information progressively unfolds, the specific moment in which the most significant influence of prior information on action anticipation becomes apparent.

In some previous studies, the predominant approach to assess this issue was to use prior information that matched the kinematic information when examining the influence of prior information on action anticipation (Farrow and Reid, 2012; Loffing and Hagemann, 2014; Broadbent et al., 2019). These studies consistently found favorable facilitative effects of prior information on action anticipation. Taking into account real-world sports scenarios, it’s important to acknowledge that opposing athletes may alter their typical actions during pivotal moments. Information acquired in advance regarding the opponent’s prior tendencies may clash with currently perceived action information. The alignment or divergence between these two types of information may impact the influence of prior information and its associated cognitive processing characteristics, particularly during crucial moments when prior information influences action anticipation. However, this hypothesis requires validation through pertinent research. Runswick et al. (2020) introduced the Model of Information use During Anticipation in Striking Sports (MIDASS), which predicts how various information sources influence accurate sports anticipation by emphasizing the alignment of information with actual outcomes. This model suggests that the impact of information sources can change over time, with early anticipation relying more on prior information and later predictions incorporating kinematic data. Anticipation performance improves when both sources align but suffers when they do not (Runswick et al., 2019). The overall performance depends on information prioritization, source reliability, and timing in the anticipation process. Therefore, the second issue that needs exploration is how prior information conflicting with kinematic information influences action anticipation performance and its cognitive processing as kinematic information progressively unfolds.

Kok et al. (2012) employed straightforward auditory and visual grating stimuli as prior and kinematic information materials, respectively. Their research revealed that predictions generated from prior information diminished visual perceptual processes related to kinematic information in individuals, potentially influencing the allocation of attentional resources toward kinematic information. Event-related potential (ERP) boasts exceptional high temporal resolution, rendering it ideal for capturing the rapid progression of sensory, cognitive, and motor processing during anticipation (Costa et al., 2023), although only a limited number of studies have investigated this issue using ERPs. In earlier research, our team extended these findings by utilizing ERPs to investigate, for the first time, the impact of congruent and incongruent prior information on brain dynamics during the action anticipation. The results provided evidence confirming one of the assumptions of the MIDASS model, namely, that action anticipation performance increased in the congruent condition and decreased in the incongruent condition. Additionally, it was observed that the amplitudes of the early ERP N1 (~100 ms) and N2 (~200 ms) components induced by the incongruent condition were significantly larger than those in the congruent condition (Wang et al., 2019a). The N1 component, evoked by an anticipation task in racket sports, is linked to early sensory and perceptual processes (Di Russo et al., 2019). Meanwhile, the N2 component is associated with processes involving inhibiting useless or conflicting information (Liu et al., 2017). These findings reflect how the congruence between the two types of information influenced early attention processes and conflict monitoring processes during the action anticipation process. However, that study did not consider the brain dynamics of anticipatory processing at critical time points when prior information influences action anticipation. That study also did not observe an allocation of attentional resources, which prior research suggests is reflected in the P3 component (Kok et al., 2012).

To tackle these aforementioned issues, the present study implemented an anticipation task involving penalty kick actions. This task provided prior information in the form of penalty direction probabilities to both experienced soccer players and to non-athletes. Drawing on the discovery made by Tomeo et al. (2013) that soccer players excel at making precise action anticipations based on kinematic information preceding the ball contact moment, as well as on the early influence of prior information as outlined by the MIDASS model, we employed a temporal occlusion paradigm in Experiment 1. This paradigm allowed us to interrupt penalty kick action videos at three distinct moments from the onset, all centered around the ball contact instant, with the aim of pinpointing the optimal timing for the impact of prior information. In Experiment 2, we conducted an assessment of ERPs to delve into the cognitive processing characteristics at this critical juncture. Our specific focus was on comprehending how prior information affected athletes’ anticipation of actions, particularly when there was a conflict between prior information and the currently available kinematic information. Drawing from the literature and the MIDASS model, our hypothesis posits two key points: first, that the pivotal time for the influence of prior information on action anticipation occurs in the early stages before the ball contacts the foot; second, at this critical juncture, congruent prior information has an advantageous effect on the anticipatory performance, manifesting as heightened cognitive processing efficiency and characterized by a reduced allocation of visual attention resources to kinematic information. Conversely, incongruent prior information produces the opposite effect, leading to increased early attention directed toward kinematic information and heightened conflict monitoring.

## 2 Experiment 1: key time points when prior information affects action anticipation in soccer goalkeepers

### 2.1 Materials and methods

#### 2.1.1 Participants

A total of 17 semi-elite soccer goalkeepers recruited from university soccer teams participated in Experiment 1 as the athlete group (Swann et al., 2015), and 18 college students with no related sports experience were recruited from Shanghai University of Sport to form the non-athlete group. The minimum sample size was estimated from a prior power analysis using G*Power (power = 0.80, alpha = 0.05, $\eta_{p}^{2}$ = 0.25) (Faul et al., 2009; Wang et al., 2019a; Costa et al., 2023). Eligibility criteria for participants were defined as follows: (a) soccer goalkeepers had been engaged in soccer-specific training for at least 5 years, (b) with practice ≥4 days a week and ≥ 3 h each practice session during the last 3 years, (c) and were qualified as a national player at the second level or above; (d) all participants were in good health, with normal vision or corrected vision, and were right-handed/footed. After the experiment, all participants received a small compensation for their participation. The experimental procedures were approved by the Ethics Committee of the Shanghai University of Sport.

#### 2.1.2 Experimental materials

The experimental material comprised a recorded penalty kick video, with a professional penalty kick player shooting with the right foot. A Canon 1D camera with video capability (resolution 1920 × 1080, 23 frames/s) was placed in the center of a standard soccer goal, 9.15 m from the penalty spot, and 1.65 m above the ground, simulating the perspective of a goalkeeper attempting to save the ball. During the recording, the penalty kick player initially stood 3.5 m to the right behind the penalty spot and then ran up and kicked the ball toward one of the four corners of the goal (upper left, lower left, upper right, and lower right). Before recording, the player was told to treat each shot as though it were occurring during a competition and thus to attempt to score, but without making any fake actions or giving any directional information to the observer. The final video that met those requirements was retained, with the ball shooting into the goal at the top left of 29 segments, bottom left of 40 segments, top right of 24 segments, and bottom right of 29 segments.

All the recorded videos were post-processed. First, Adobe Premiere Software (Adobe Systems Incorporated, USA) was used to convert each video to a continuous video at 20 frames/s. Using a temporal occlusion paradigm, three time points were selected for video truncation: Time (T), which comprised a total of 42 frames from start to the foot-ball contact and included the frame in which the foot contacted the ball; T-1, which comprised 41 frames from the start until one frame before the foot-ball contact (i.e., up to 50 ms before the foot-ball contact); and T-2, which comprised 40 frames from the start up to 100 ms before the foot-ball contact. To eliminate the interference from head movement cues toward the goal direction during the early preparation phase of the player’s shot in the videos, the first 26 frames were excluded. Consequently, there are 16 frames for the T condition, 15 frames for the T-1 condition, and 14 frames for the T-2 condition. The amount of kinematic information contained in those three time blocking point conditions was T > T-1 > T-2. We then used Photoshop (Adobe System Incorporated, USA) to blur the player’s face in each frame to avoid providing the action direction of the player’s head and eyes, which may indicate the shooting direction (Wang et al., 2019a,b). Each image was cropped to a uniform size of 1024 × 576 pixels. Finally, MATLAB R2011a (MathWorks, USA) was used to export the processed images into a video in Audio Video Interleave (.avi) format (Table 1).

**Table 1:** Video materials.

Stimulus abbreviation	Time occlusion point	Total frames (starting from the run up to the ball)	Video duration (milliseconds)	Amount of kinematic information contained
T-2	2 Frames before foot-ball contact	14	700	Small
T-1	1 Frame before foot-ball contact	15	750	Moderate
T	Foot-ball contact	16	800	Large

#### 2.1.3 Experimental task and procedure

Current kinematic information was considered all information that was contained in the shooting action of the player in the video. The prior information was data given to the participant on the probability of the player’s shooting direction. Because the purpose of the experiment was to distinguish the effects of the prior information itself and the impact of the effectiveness of the prior information on action anticipation performance, this prior information was presented in the form of guidance language with three conditions: with directional information, without directional information, and without prior information.

The condition without prior information meant that no information about the probability of the shooting direction was presented to the participants before the task. Thus, the action anticipation of the participants was processed only by using the current body kinematics information of the shooting player in the video. In the condition without directional information, before the task, the probability of the player’s shooting direction in the ensuing video materials was presented to the participants, but the probability of kicking the ball was the same for all four locations of the goal. In the condition that contained directional information, participants were told the probability of player kicking the ball toward a specific location of the goal in 75% of the trials as the ball flew toward the goal.

All participants were required to complete the action anticipation task with one block each of all three experimental conditions. Before each block, the instruction for the prior information condition was presented. The instruction for the condition without prior information was, “In the next set of videos, there is no information available. Please predict the direction of the ball trajectory based on the presented visual information.” The instruction for the condition without directional information was, “In the next set of videos, the likelihood of shooting in four directions is the same: upper left, lower left, upper right, and lower right.” The instruction for the condition with directional information was, “The majority of the shooting directions lean toward the upper-left corner.” The order in which the blocks with the three prior information conditions was presented was balanced among the participants to eliminate confounding by learning or sequence effects. Participants were asked to predict the final flight direction of the ball in each trial based on the verbal guidance and the player’s actions in the video by pressing the A key on a computer keyboard to indicate the top left, X key to indicate bottom left, L key to indicate top right, and M key to indicate bottom right. After the participants understood the guidance, they pressed any key to start the formal action anticipation task. Both conditions without prior and directional information blocks included 60 trials, with equal proportions of kicks in all four directions and three occlusion points. While in the condition with directional information block, 75% of the kicks in the 60 trials were in the upper-left corner. In each trial, a fixation (attention) point was first presented for 400 ms, followed by a video of the penalty kick in the blocked video. Participants were asked to predict the direction of the ball trajectory within 3 s. The experimental task was designed and conducted using E-Prime software (version 2.0, PST, Inc.; Pittsburgh, PA, USA).

#### 2.1.4 Data and statistical analyses

We calculated the percentage of correct responses (accuracy) and response time (RT) for each experimental condition. Both accuracy and RT were assessed using a three-way analysis of variance (ANOVA), with group (athletes vs. non-athletes) as the between-subject factor, prior information (without prior information, without directional information, and with directional information) and the amount of kinematic information (T, T-1, and T-2) as the within-subject factors to explore the impact of prior information on action anticipation given three levels of current kinematic information among soccer goalkeepers. SPSS 20.0 was employed for statistical analysis, and the Greenhouse–Geisser method was used to correct the freedoms degree and value of *p* for analyses that did not satisfy the sphericity test. The Bonferroni method was used for *post hoc* testing, and the results were considered statistically significant when *p* < 0.05.

### 2.2 Results

**Figure 1:**
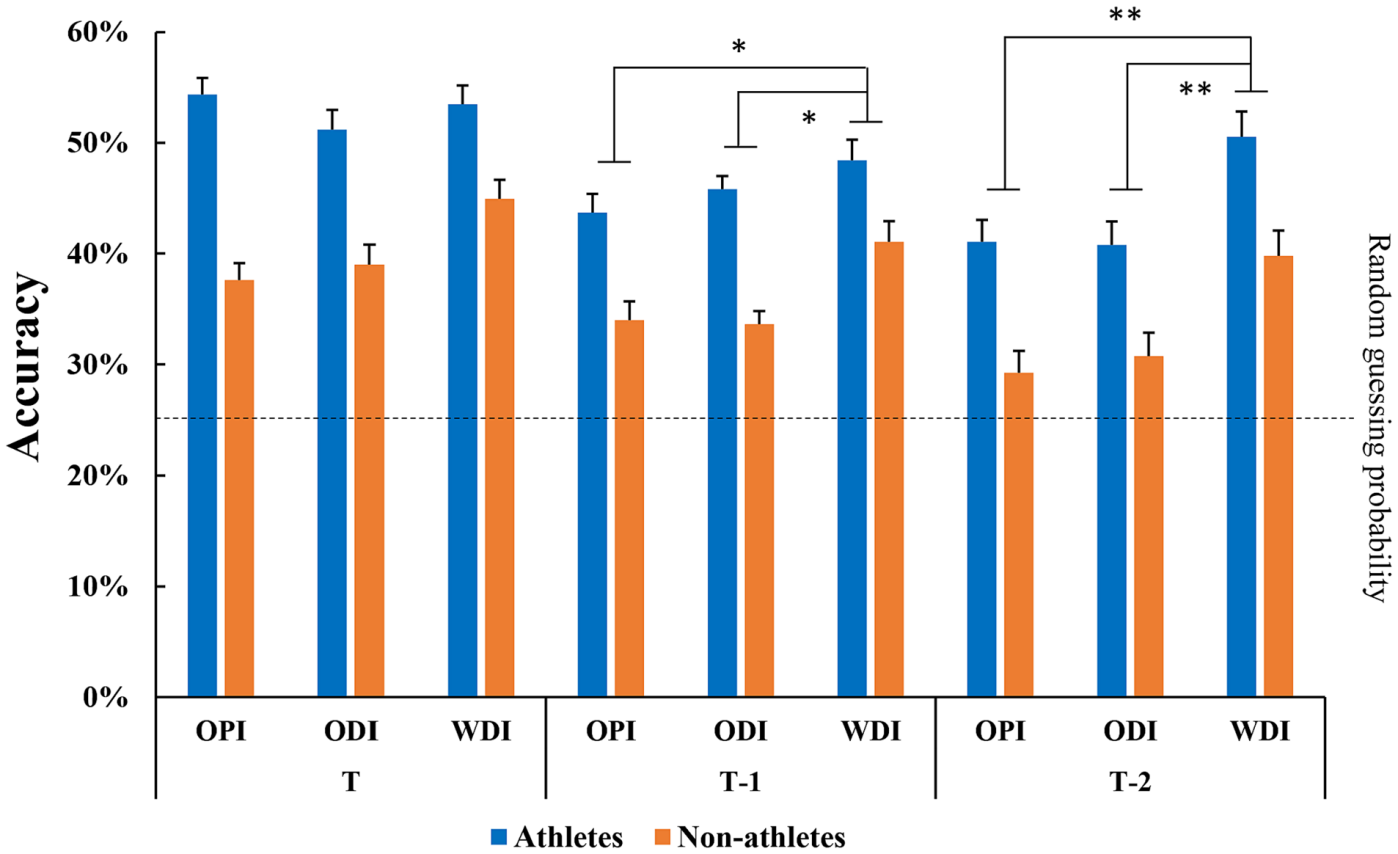
Comparisons of action anticipation accuracy between the athlete and non-athlete groups for three amounts of kinematic information and three prior information conditions in Experiment 1. The amount of kinematic information was considered large (T), moderate (T-1), or small (T-2). Prior information was divided into three conditions: without prior information (OPI), without directional information (ODI), and with directional information (WDI). * represents *p* < 0.05; **p < 0.01 for the indicated comparisons.

The results of a 2 (group) × 3 (prior information) × 3 (kinematic information) ANOVA showed that the main effect of group was significant (*F* (1, 33) = 128.04, *p* < 0.001, $\eta_{p}^{2}$ = 0.795). The *post hoc* comparisons indicated that the goalkeepers’ anticipation accuracy was significantly higher than that of the non-athlete group. The interaction between prior and kinematic information was significant (*F* (4, 132) = 2.49, *p* = 0.046, $\eta_{p}^{2}$ = 0.070). A simple effects test indicated that the anticipatory accuracy with directional information given was significantly higher than that when no prior information was given or when prior information without directional information was given (*p* < 0.001) in both T-1 (*p*’s ≤ 0.034) and T-2 (*p*’s < 0.001) conditions. However, in the T condition, there were no significant differences among the three types of prior information conditions (*p*’s ≥ 0.078) (Figure 1). The three-way interaction was not significant (*F* (4, 132) = 1.08, *p* = 0.369, $\eta_{p}^{2}$ = 0.032).

The results of the same ANOVA with RT as the dependent variable indicated a significant main effect only for kinematic information (*F* (2, 132) = 7.76, *p* = 0.001, $\eta_{p}^{2}$ = 0.190). The RT in the T condition was higher than that in the T-1 (*p* = 0.005) or T-2 condition (*p* = 0.020). The other main effects and interactions were not significant (*p*’s ≥ 0.442).

### 2.3 Experiment 1 discussion

The aim of Experiment 1 was to investigate the impact of prior information on action anticipation in soccer goalkeepers and to examine how this effect varied under different levels of kinematic information. To the best of our knowledge, there has been relatively little research examining the influence of prior information on the penalty kick prediction performance of goalkeepers and its interaction with the level of kinematic information, likely due to the limited number of semi-elite soccer goalkeepers (only approximately 20 available) (Savelsbergh et al., 2002; Navia Manzano et al., 2013; Tomeo et al., 2013; Mann et al., 2014; Wang et al., 2019a; Murta et al., 2022). A strength of the present study was that participants were instructed to anticipate the ball trajectory in four directions, as opposed to just one direction, thereby aligning the task more closely with real-world goalkeeping scenarios to enhance the validity of the action anticipation paradigm (Navia Manzano et al., 2013).

Experiment 1 provided evidence confirming the advantage of expertise that has been reported in many previous studies, finding that goalkeepers outperformed non-athletes in anticipation accuracy (Aglioti et al., 2008; Smith, 2016; Wang et al., 2022; Costa et al., 2023). Furthermore, this study showed that participants demonstrated significantly improved accuracy in predicting penalty kick directions when provided with directional information, in contrast to conditions where no prior information or directional information was available. This effect was particularly pronounced when kinematic information was occluded 50 and 100 ms before the ball-to-foot contact. This finding suggested that prior information about the kicker’s action tendencies had a greater impact on anticipatory performance in the presence of limited kinematic information, compared with when the kicker exhibited ample kinematic information. Gredin et al. (2021) reported similar findings in their study in which action anticipation during progressive temporal occlusion revealed that the semi-elite soccer players relied more on the opponent’s action tendencies in the early occlusion condition compared with the later occlusion condition. The results also aligned with the assumptions of the MIDASS model, which suggests that early action anticipation relies more on prior information (Runswick et al., 2020). This preferred reliance is primarily because the kinematic information that appears early in the process is not complete, and the provided information is insufficient for accurately predicting the action outcome. Thus, in comparison, prior information proves to be more reliable.

We did not observe a significant difference in anticipatory performance between the conditions without prior information and without directional information. This finding suggested that, even when participants were aware that the kicker’s probabilities of shooting in each of the four directions were identical, they could not improve their anticipatory performance by adopting a consistent response strategy across all four keys. To some extent, it dismisses the possibility that the facilitating effect of prior information with directional tendencies on action anticipation is due to participants calculating response probabilities. The finding underscores that only prior information with directional tendencies could enhance anticipatory performance.

Based on the findings from Experiment 1, we established that the maximum enhancement in anticipatory performance occurred when kinematic information was presented up to at least 50 ms before the ball-to-foot contact, in conjunction with prior directional information. Building on this, Experiment 2 utilized soccer penalty kick action videos at this occlusion time point as stimulus material. The aim of Experiment 2 was to delve into the underlying brain dynamics, with the intention of elucidating the adaptive cognitive-motor behavior exhibited by highly trained soccer players at the point when their anticipatory performance was most influenced by prior information. Specifically, we conducted a comparison of ERPs in response to congruent and incongruent events by manipulating the congruency of prior information and kinematic information.

## 3 Experiment 2: the impact of congruent and incongruent prior information on anticipatory brain dynamics

### 3.1 Materials and methods

#### 3.1.1 Participants

In Experiment 2, in response to the scarcity of available professional soccer goalkeepers, we recruited 27 soccer players from the Shanghai Shanggang soccer reserve team. All athletes had a minimum of 5 years of soccer training, held a sports ranking at the national second-class level or higher, and possessed several years of goalkeeping experience. We also recruited a non-athlete group comprising 27 college students devoid of any prior sports training experience. The eligibility criteria for participants was similar to Experiment 1. No individual participated in both experiments.

#### 3.1.2 Experimental materials

A professional soccer player who was not participating in the study recorded the ball-shooting video. The video recording and processing were the same as in Experiment 1. Based on the results of Experiment 1, the temporal blocking points T-1 and T-2 were selected, and the ratio of the video presentation at the two blocking points was balanced within the groups.

#### 3.1.3 Experimental task and procedure

The action anticipation task had three conditions: neutral, congruent, and incongruent. The neutral condition was identical to the condition without directional information in Experiment 1, while the congruent and incongruent conditions entailed prior information with a directional bias. Each condition was treated as a block with instructions providing the relevant prior information. For the neutral condition, participants were instructed as follows: “In the next set of videos, the likelihood of shooting in four directions is the same: upper left, lower left, upper right, and lower right.” In both the congruent and incongruent conditions, participants received the same instructions: “In the next set of videos, the majority of shots will be aimed toward the upper-left corner.” The difference between the congruent and incongruent conditions was in the distribution of trials. In the congruent condition, 75% of the 60 trials in the block indeed featured shots aimed at the upper-left corner of the goal. In contrast, the incongruent condition retained the tendencies of the neutral condition, with an equal probability of all four goal directions (25%) for shot occurrence.

During each trial of the task, a fixation point was first presented for 500 ms, followed by a blocked penalty video. The participants were required to predict the direction of the ball trajectory within 3 s. A random interval of 1,000–1,500 ms was given between the trials.

#### 3.1.4 ERP data recording

Brain electrical activity was recorded from 64 Ag/AgCl electrodes arranged according to the International 10–20 System, with a sampling rate of 500 Hz (Brain Products GmbH, Munich, Germany). An electrode for the vertical electrooculogram was placed below the left eye, and for the horizontal electrooculogram, lateral to the right eye. Electroencephalography (EEG) activity was online referenced to the FCz site; the AFz site served as the ground electrode. All electrode impedances were maintained below 10 kΩ.

#### 3.1.5 Data analysis

For the behavioral data, we calculated the accuracy and RT for each prior information condition. The data were entered into a two-way ANOVA, with group (athletes vs. non-athletes) as the between-subject factor, and prior information condition (neutral, congruent, and incongruent) as the within-subject factor.

ERP data were analyzed offline using BrainVision Analyzer software (version 2.1; Brain Products, Gilching, Germany). The average potential of the bilateral mastoid (TP9 and TP10) was used as a reference. Oculomotor artifacts of the EEG signal were removed using a regression procedure implemented in the Analyzer software, and 50-Hz main interference was also removed. The data were low-pass filtered at 30 Hz and high-pass filtered at 0.1 Hz (the slope was 24 dB/octave). Each condition was segmented separately. With the video onset taken as the 0 point, the first 200–800 ms after onset was selected, and the data outside the range of ±100 μV was eliminated. Typical electrode points (see results section) and time windows for each component were determined based on the average waveform maps, brain topography maps assessed by visual inspection, and previous literature (Wang et al., 2019a). The time windows were centered on each of these peaks. The windows width was chosen to include all individual peaks of the same component. No latencies differences were evidenced among peaks of different conditions or groups; therefore, the following ERP components were calculated as the mean amplitude in selected time windows (Costa et al., 2023), as follows: the N1 in the 90–140 ms; the N2 in the 220–350 ms; the P3 in the 460–550 ms. For the amplitude of each ERP component, a 2 (group: athletes and non-athletes) × 3 (prior information condition: neutral, congruent, and incongruent) repeated-measures ANOVA was conducted. The brain-behavior correlation analyses were also performed between the differences in ERP components’ amplitudes (Incongruent-Congruent) and the same differences in the anticipatory response efficiency for each group.

SPSS 20.0 software was used for statistical analysis. The Greenhouse–Geisser method was used to correct the degrees of freedom and *p*-values for statistics that did not satisfy the sphericity test. The Bonferroni method was used for *post hoc* tests, with the results considered significant when *p* < 0.05.

### 3.2 Results

#### 3.2.1 Behavioral results

With accuracy as the dependent variable, a 2 (group) × 3 (prior information condition) repeated-measures ANOVA was performed. The results indicated that the main effect of group was significant (*F* (1, 52) = 36.68, *p* < 0.001, $\eta_{p}^{2}$ = 0.414). *Post hoc* comparisons found that anticipation accuracy in the athletes was significantly higher than that in the non-athlete group. The main effect of condition was also significant (*F* (2, 104) = 17.89, *p* < 0.001, $\eta_{p}^{2}$ = 0.256). *Post hoc* comparisons indicated that anticipation accuracy under congruent conditions was significantly higher than that under both neutral (*p* < 0.001) and incongruent (*p* = 0.001) conditions. The interaction between condition and group was not statistically significant (*F* (2, 104) = 0.19, *p* = 0.824, $\eta_{p}^{2}$ = 0.004).

**Figure 2:**
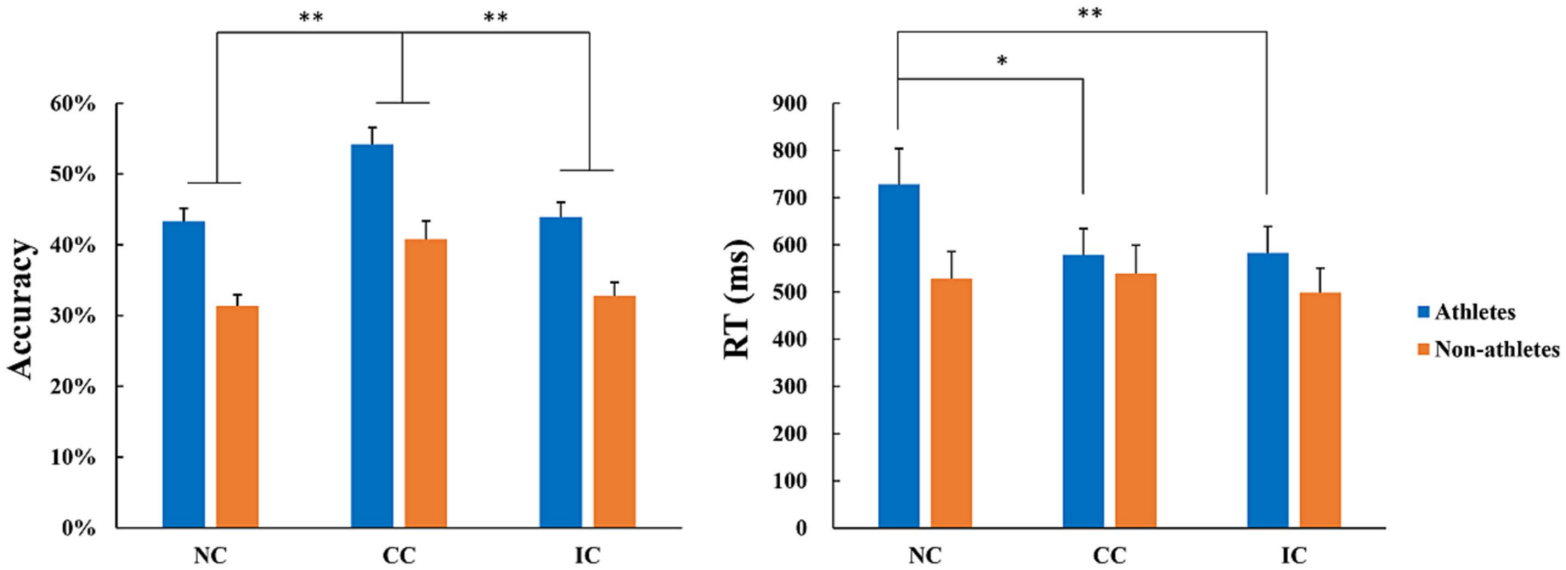
Comparisons of action anticipation accuracy and RT between the athlete and non-athlete groups under three prior information conditions in Experiment 2. Prior information was divided into three conditions: neutral (NC), congruent (CC), and incongruent condition (IC). * represents *p* < 0.05; **p < 0.01 for the indicated comparisons.

The repeated-measures ANOVA with RT as the dependent variable found that the main effect of group was not significant (*F* (1, 52) = 1.94, *p* = 0.170, $\eta_{p}^{2}$ = 0.036), but the main effect of condition (*F* (2, 104) = 4.78, *p* = 0.010, $\eta_{p}^{2}$ = 0.084) and the interaction between the two (*F* (2, 104) = 3.83, *p* = 0.025, $\eta_{p}^{2}$ = 0.069) were significant. Simple effects analysis results showed that the response speed of the athletes under the neutral condition was slower than that under both the congruent (*p* = 0.012) and incongruent (*p* = 0.009) conditions. For the non-athlete group, there was no significant difference in RT for the three conditions (Figure 2).

#### 3.2.2 ERP results

**Figure 3:**
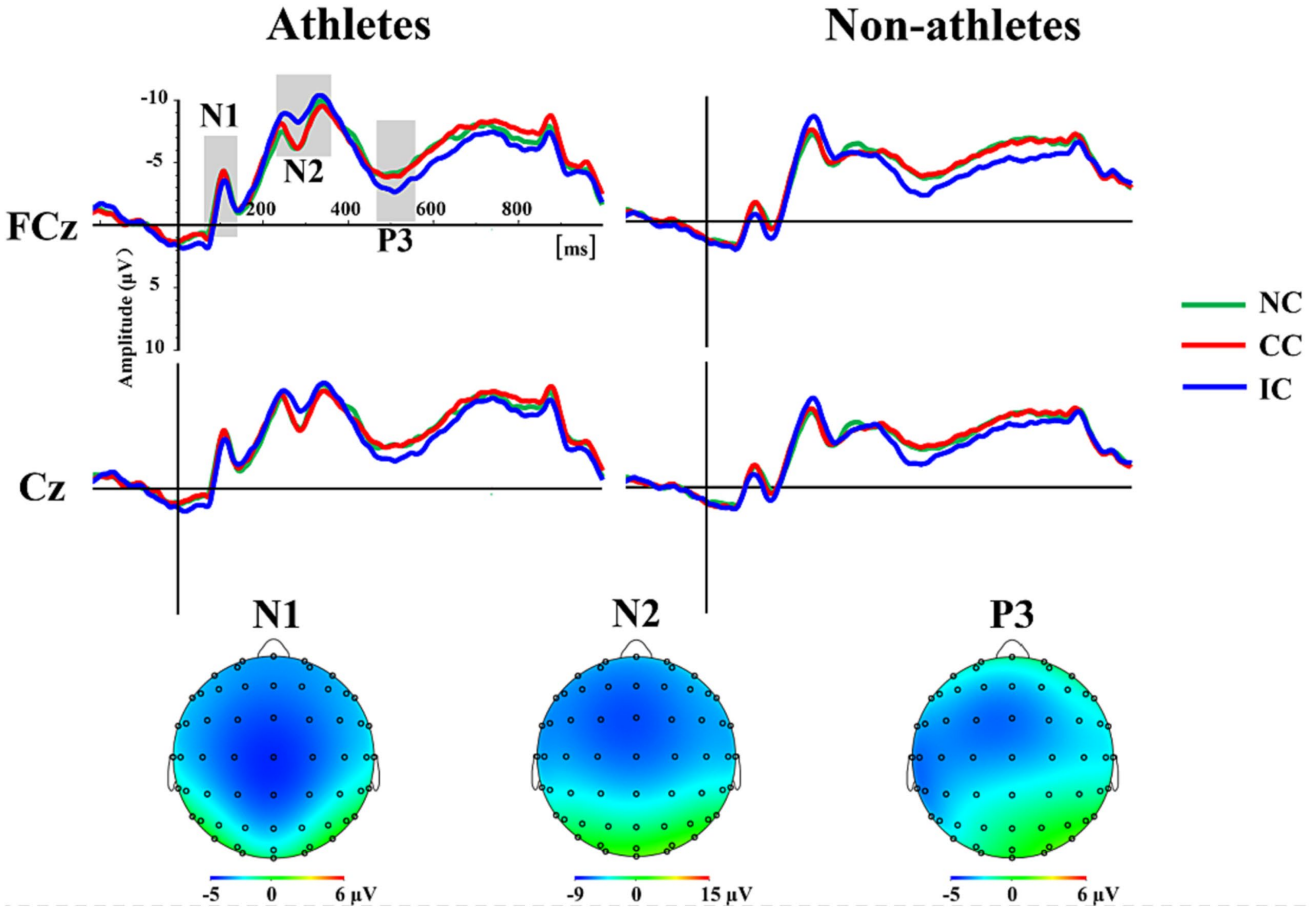
The grand-averaged ERP waveforms of three prior information conditions at the FCz and Cz, as well as the scalp topographies of the N1, N2 and P3 components. Prior information was divided into three conditions: neutral (NC), congruent (CC), and incongruent condition (IC).

**Figure 4:**
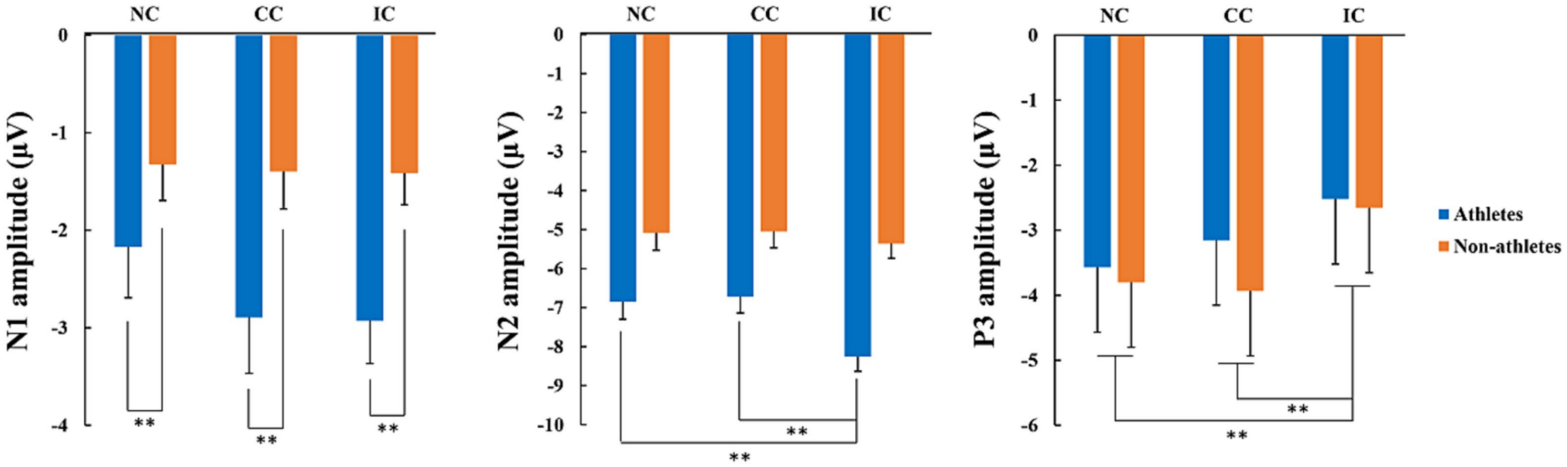
Comparisons of N1, N2 and P3 amplitudes between the athlete and non-athlete groups under three prior information conditions. Prior information was divided into three conditions: neutral (NC), congruent (CC), and incongruent condition (IC). ** represents *p* < 0.01 for the indicated comparisons.

Assessing total average waveforms (Figure 3), we identified distinct N1, N2, and P3 components. For the N1 component, the mean amplitude of the five electrode points (FCz, Cz, CPz, C1, and C2) based on the topographical distribution of grand-averaged ERP activity was considered the dependent variable in a 2 (group) × 3 (prior information condition) repeated-measures ANOVA. We found that the main effect of group was significant (*F* (1, 52) = 5.06, *p =* 0.029, $\eta_{p}^{2}$ = 0.089), and the N1 amplitude in the athletes was significantly higher than that in the non-athlete group. The main effect of prior information condition was not significant (*F* (2, 104) = 2.29, *p* = 0.107, $\eta_{p}^{2}$ = 0.042), nor was the interaction between group and condition (*F* (2, 104) = 1.47, *p* = 0.235, $\eta_{p}^{2}$ = 0.027) (Figure 4).

For the N2 and P3 ERP components, the mean amplitude of the five electrode points (Fz, FCz, Cz, FC1, and FC2) was considered a dependent variable for the 2 (group) × 3 (prior information condition) repeated-measures ANOVA. For the N2 component, the results indicated that the interaction between group and condition was significant (*F* (2, 104) = 3.89, *p* = 0.023, $\eta_{p}^{2}$ = 0.070). The results of a simple effects analysis indicated that the N2 amplitude in the athletes in the incongruent condition was significantly higher than that in both neutral (*p* < 0.001) and congruent (*p* < 0.001) conditions. However, there was no significant difference in the N2 amplitude among the three conditions in the non-athletes.

For the P3 ERP component, the results indicated that the main effect of group was not significant (*F* (1, 52) = 0.31, *p* = 0.581, $\eta_{p}^{2}$ = 0.006), whereas the main effect of condition was significant (*F* (2, 104) = 10.06, *p* < 0.001, $\eta_{p}^{2}$ = 0.162). *Post hoc* comparative analysis indicated that the P3 amplitude induced by the incongruent condition was significantly higher than that induced by both neutral (*p* = 0.001) and congruent (*p* = 0.002) conditions. The interaction between group and condition was not statistically significant (*F* (2, 104) = 0.86, *p* = 0.427, $\eta_{p}^{2}$ = 0.016).

The brain-behavior correlation analyses showed no significant correlations between ERP components’ amplitudes and anticipatory performance for both groups (*p* ≤ 0.077).

### 3.3 Experiment 2 discussion

In Experiment 2, we investigated the impact of prior information on brain dynamics during action anticipation, particularly when there was inconsistency between prior information and kinematic information, at the time point where the effects of prior information were most pronounced as determined in Experiment 1. Consistent with Experiment 1, the results of Experiment 2 reaffirmed the expert advantage of the soccer players and further indicated that the accuracy of the athletes was consistently better than that of the non-athlete group across all congruency conditions. This finding indicated that athletes were better equipped than non-athletes to utilize and integrate prior and kinematic information to effectively carry out action anticipation (Gredin et al., 2018). We also found that anticipatory performance was increased when anticipating kicks congruent with prior directional tendencies compared with that which was incongruent with prior information. However, we did not observe a decrease in anticipatory accuracy in the incongruent condition compared with the neutral condition. These findings further corroborated the utilization of prior information during action anticipation (Gredin et al., 2023), with the caveat that only prior information aligned with kinematic information enhanced anticipatory accuracy.

When considered alongside the RT results, the interaction between group and condition indicated that the presence of directional tendencies in prior information improved the reaction speed of athletes, regardless of its congruence with kinematic information. However, the reaction speeds of the non-athlete group did not exhibit significant differences across the three conditions. This disagreement in RTs underscored the unique advantage of athletes in rapidly processing and utilizing prior information (Hülsdünker et al., 2019), emphasizing their heightened cognitive abilities in the domain of action anticipation (Yarrow et al., 2009). This is a relatively rare finding in the existing literature on how prior information facilitates action anticipation (Murphy et al., 2018; Runswick et al., 2018; Gredin et al., 2019; Wang et al., 2019a). It may suggest that the reason many studies have consistently observed faster RTs in athletes is due to their accustomed use of various information sources to enhance their response efficiency (Mankowska et al., 2015; Hülsdünker et al., 2019; Presta et al., 2021), especially in fast-paced ball sports that demand rapid RTs (Singer, 2000; Triolet et al., 2013).

Another notable finding in Experiment 2 is that precisely when prior information exerted its most substantial impact on action anticipation, athletes and novices exhibited comparable levels of cognitive processing, regardless of whether they were confronted with conflicting information from various sources. Regarding the early ERP components, we observed significantly higher N1 amplitudes in the semi-elite athletes compared with the non-athlete group. Given that the N1 amplitude reflects selective attention to stimulus properties (Moore et al., 2017), we suggest that athletes, when presented with prior information, focused their attention on the details of the kinematic information to better assess the consistency between these two types of information. Previous research has found that athletes employ different response strategies when confronted with conflicting situations compared with expected scenarios (Lu et al., 2020). The Predicted Response Outcome (PRO) model posits that cognitive preparatory processes based on brain neural activity are diminished when information is consistent and enhanced when information is inconsistent (Alexander and Brown, 2011). In the process of monitoring the consistency between these two types of information, athletes invested greater early attention, enabling them to better discern the information’s congruence. The increased P3 amplitude observed in the present study under incongruent conditions further corroborated the theories associated with the PRO model and indicated that when confronted with inconsistent information, the P3 amplitude, which reflects the allocation of attentional resources (Liu et al., 2017; Wang et al., 2022), became larger.

The N2 amplitude induced in the athletes in the incongruent condition was markedly higher than that in both the congruent and neutral conditions, with no such difference observed in the non-athlete group. This finding provides strong support for the conflict interpretation of the N2 component because the N2 amplitude was previously shown to increase for an unexpected or incongruent stimulus (Donkers and Van Boxtel, 2004; Purmann et al., 2011). These results indicated that the athletes were able to distinguish the consistency between prior information and kinematic information, with a more pronounced N2 amplitude in conflicting situations. These findings are consistent with our previous research (Wang et al., 2019a) in which we utilized a basic arrow direction to represent prior information within a single trial. These findings together indicated that across different experimental designs, the N2 component consistently served as a reliable indicator of information conflict.

## 4 General discussion

The aim of this study was to investigate the impact of the congruence between prior information and kinematic information on the processing of action anticipation at the moment when the effect of prior information was most pronounced. The findings of the two experiments together suggested that even when the impact of prior information was at its peak, athletes continued to focus on incomplete kinematic information. This strategic attention allocation is crucial for detecting any conflict between the two information sources, ultimately leading to more accurate and faster action anticipation.

The results of Experiment 1 were consistent with our first hypothesis. That is, we found that prior information had the greatest impact on action anticipation when presented at least 50 ms before the ball-to-foot contact. Building on this outcome, Experiment 2 utilized ERPs to test the second hypothesis. Our findings indicated that early visual attention processes were not influenced by conflicting information. However, we observed heightened engagement of conflict monitoring and late-stage attentional resource allocation processes in response to the incongruent condition. The results of this study hold significant importance in supporting not only our second hypothesis but also the hypothesis put forth by the MIDASS model, which suggests that the reliance on these two types of information may dynamically change with the completeness of kinematic information. Furthermore, these findings serve to augment the model’s explanatory power regarding how the congruency of information can exert differing effects on anticipatory performance during the integration of these two information sources.

While certain findings in this study align with prior research, such as the observed superior action anticipation efficiency of athletes compared with non-athletes and their increased sensitivity in identifying congruency between prior and kinematic information (Jin et al., 2011; Wu et al., 2013; Wang et al., 2019a), this study also extends these results. Through deliberate manipulation of the effects of prior information, we accentuated its influence on the action anticipation process, particularly in relation to attention processes concerning kinematic information. This holds pivotal significance in comprehending the mechanisms at play when integrating diverse information sources to enhance reaction efficiency, thereby standing as a noteworthy highlight of this study.

The results from Experiment 1 notably showed that only prior information incorporating directional tendencies enhanced anticipatory performance when kinematic information was scarce. By contrast, prior information lacking directional biases proved to be ineffective and failed to elicit improvements in RT. These findings highlight the crucial importance of probabilistic prior information that exhibits a bias toward specific outcomes in bolstering athletes’ performance in action anticipation—a facet that has been relatively understudied in comparison with other aspects of research in this field (Gredin et al., 2018, 2019, 2023; Broadbent et al., 2019). We can use the study by Broadbent et al. (2019) as an example to interpret the results we have found from an anxiety perspective. Their research aimed to investigate the influence of anxiety on athletes utilizing prior information for action anticipation. However, their results did not reveal an interaction between anxiety levels in soccer players and the congruency between prior information and kinematic information regarding their impact on anticipatory performance. The absence of interaction may potentially stem from the comparison with a “no priors” condition, where the lack of prior information could impact anxiety-related performance. Broadbent et al.’s study only encompassed two conditions—no information and biased prior information—omitting a condition with unbiased prior information. The introduction of additional information might alleviate performance effects associated with anxiety (Charpentier et al., 2022), thereby influencing the anxiety variable’s effects. Consequently, the distinction between the “no information” and “biased prior information” conditions in their study, besides biased information presence, may introduce an unrelated variable concerning information provision and its potential to induce anxiety in participants. To address this concern, it would be beneficial to consider introducing a condition involving unbiased prior information as an alternative to the “no priors” condition. Thus, in our study, both Experiment 1 and Experiment 2 included a condition with unbiased prior information. This not only helped elucidate which type of prior information effectively enhances athletes’ action prediction performance but also potentially mitigated interference effects from unrelated factors, such as anxiety.

Several limitations of the current study should be acknowledged. First, we did not enlist professional soccer goalkeepers for Experiment 2 but rather recruited soccer players with extensive goalkeeping experience. This choice was made due to the limited availability of professional goalkeepers as study participants. However, this decision may impact the conclusions drawn from Experiment 2. Nevertheless, it is noteworthy that we observed results akin to those in Experiment 1, indicating that the athlete group, compared to the non-athlete group, exhibited a higher accuracy in action prediction, attributable to their goalkeeping experience. Second, we used a traditional analysis of EEG data even for the video-elicited EEG. Future research endeavors can deepen our understanding of the assumptions made by the MIDASS model by utilizing alternative EEG data analysis techniques or incorporating additional methodologies, such as eye-tracking and functional magnetic resonance imaging (Wang et al., 2012; Bishop et al., 2013; Lu et al., 2020). Those complementary methods have the potential to provide a more thorough and detailed examination of the model’s postulations.

## 5 Conclusion

The results of this study provided support for the hypotheses presented by the MIDASS model concerning the temporal aspects and conflict dynamics involved in the integration of prior information and kinematic information during the process of action anticipation. In the presence of prior information containing directional tendencies, semi-elite soccer players exhibited an elevated level of selective attention toward the characteristics of forthcoming actions during the early phases of kinematic information processing. This heightened attention allocation served as a preparatory step for the subsequent identification of potential conflicts between kinematic and prior information, and notably, this process appeared to be more distinct in athletes compared with non-athletes. Additionally, the availability of prior information enhanced RTs only among semi-elite soccer players.

## Data availability statement

The raw data supporting the conclusions of this article will be made available by the authors, without undue reservation.

## Ethics statement

The studies involving humans were approved by Ethics Committee of the Shanghai University of Sport. The studies were conducted in accordance with the local legislation and institutional requirements. Written informed consent for participation in this study was provided by the participants’ legal guardians/next of kin. Written informed consent was obtained from the individual(s) for the publication of any potentially identifiable images or data included in this article.

## Author contributions

QJ: Data curation, Formal analysis, Methodology, Visualization, Writing – original draft. CZ: Resources, Validation, Project administration, Writing – review & editing. YW: Conceptualization, Supervision, Investigation, Software, Writing – review & editing.
